# Evidence‐Based Medicine Meets Artificial Intelligence: Reframing Critical Appraisal in Emergency Medicine

**DOI:** 10.1111/acem.70381

**Published:** 2026-07-15

**Authors:** Sangil Lee, Michael Brown, Rebekah Richards, Moira Smith

**Affiliations:** ^1^ Department of Emergency Medicine Weill Cornell Medicine New York New York USA; ^2^ Department of Emergency Medicine Michigan State University East Lansing Michigan USA; ^3^ Department of Emergency Medicine The Ohio State University Wexner Medical Center Columbus Ohio USA; ^4^ Department of Emergency Medicine University of Virginia School of Medicine Charlottesville Virginia USA

**Keywords:** artificial intelligence, critical appraisal, emergency medicine, evidence‐based medicine, large language models, medical education

Emergency physicians practice in an environment characterized by high cognitive load, diagnostic uncertainty, and rapidly evolving evidence [1]. The volume of biomedical literature continues to expand at a pace that challenges even experienced evidence‐based medicine (EBM) practitioners [2]. Although critical appraisal remains foundational to EBM, it is increasingly difficult to sustain in busy clinical and academic settings [3]. Artificial intelligence (AI), particularly large language models (LLMs), have rapidly entered this space and are beginning to reshape how clinicians interact with existing evidence [4].

The question is no longer whether AI can contribute to critical appraisal, but rather how it should be integrated responsibly into evidence synthesis, peer review, and medical education. Current discussions frequently emphasize the importance of “keeping a human in the loop” [5]. While conceptually reassuring, this framing may underestimate the degree to which AI is already capable of performing structured components of evidence appraisal [6]. A more practical framework is emerging: AI‐first, human‐adjudicated critical appraisal. In this model, AI performs the structured, rule‐based steps first, while human experts adjudicate the interpretive judgments, methodological nuance, contextual relevance, and clinical applicability that AI cannot yet make reliably. The claim is therefore not that AI should lead the entire appraisal, but that it should lead the components for which it is demonstrably suited.

Critical appraisal consists of literature identification, study screening, data extraction, risk‐of‐bias assessment, interpretation, and clinical application [6]. These tasks differ in their dependence on human judgment: AI performs best for structured, rule‐based processes, whereas clinicians retain advantages in contextual interpretation and clinical applicability [7]. This distinction increasingly mirrors modern systematic review workflows [8]. AI‐assisted platforms can now screen abstracts, extract study characteristics, summarize findings, and organize evidence into structured outputs with substantial efficiency gains [9]. Their utility is greatest when prompts are highly structured and expectations are explicit [10].

Recent empirical studies provide insight into where AI performs well and where important limitations remain. In a 2025 study published in *Annals of Internal Medicine*, investigators compared AI‐assisted and human‐only data extraction across 63 studies included in six systematic reviews [11]. AI‐assisted extraction achieved 91% accuracy compared with 89% for human‐only extraction while reducing extraction time by approximately 41 min per study [11].

However, performance appears less robust when subjective methodological judgment is required. A 2025 study published in *Cochrane Evidence Synthesis and Methods* evaluated ChatGPT‐assisted risk‐of‐bias assessments using the Cochrane RoB 1.0 framework [12]. Agreement between AI and human reviewers for overall risk‐of‐bias judgments was approximately 50%, although agreement was substantially higher for more objective domains such as randomization methods [12].

AI‐assisted appraisal may accelerate evidence synthesis without sacrificing methodological rigor. Clinicians can rapidly summarize new evidence before deeper review, researchers can streamline systematic review workflows, and educators can use AI‐generated appraisals to teach critical evaluation rather than rote appraisal.

Importantly, disagreement between AI outputs and human reviewers should not necessarily be viewed as failure. Rather, discrepancies may identify precisely where deeper methodological scrutiny is needed. For example, AI may classify a domain as unclear because reporting is ambiguous, whereas an experienced reviewer may draw on additional methodological context. In this framework, AI becomes a cognitive scaffold that accelerates information processing while simultaneously highlighting areas requiring expert adjudication.

This evolving workflow also shifts the educational emphasis of EBM training. Historically, critical appraisal education has emphasized procedural completion of checklists and structured frameworks. While these skills remain essential, AI may increasingly automate many mechanical components of appraisal. The comparative advantage of clinicians may therefore shift toward interpretation, contextualization, and recognition of subtle methodological limitations that are not easily reducible to rules or templates. In this sense, AI does not diminish the importance of EBM expertise; rather, it may increase the importance of higher‐order methodological reasoning.

Despite its promise, AI‐assisted critical appraisal carries important risks. Large language models may generate plausible but incorrect interpretations, a phenomenon commonly referred to as hallucination or confabulation. Over‐reliance on AI outputs may encourage superficial review and automation bias, particularly among inexperienced clinicians. AI systems may also reproduce or amplify biases embedded within their training data. Furthermore, confidentiality concerns remain highly relevant in peer review settings, as many journals prohibit uploading unpublished manuscripts into external AI systems due to concerns regarding copyright, privacy, and intellectual property [13].

These limitations reinforce the importance of active human adjudication rather than passive oversight. The future of critical appraisal is unlikely to be human‐only or AI‐only. Instead, the emerging model is collaborative: AI expands capacity by automating structured cognitive tasks, while clinicians ensure validity through interpretation, skepticism, contextual judgment, and clinical reasoning. As emphasized by Hopson and others, uncertainty is an inherent feature of medical decision making rather than a problem to be eliminated. AI can organize evidence and highlight methodological concerns, but it cannot determine how persistent uncertainty should be interpreted, communicated, and incorporated into patient‐centered decisions. Those judgments require human expertise, experience, and professional accountability.

AI‐assisted critical appraisal represents a natural evolution of evidence‐based medicine by expanding clinicians' capacity for structured evidence synthesis. However, AI cannot determine whether evidence is trustworthy, clinically meaningful, or applicable to individual patients. An AI‐first, human‐adjudicated framework therefore offers a practical model that combines computational efficiency with human judgment and warrants prospective evaluation.

## Author Contributions

S.L. conceived the manuscript and drafted the initial version using the generative AI. M.B., R.R., and M.S. contributed to manuscript development, critical revision of the content, and approval of the final manuscript.

## Funding

The authors have nothing to report.

## Conflicts of Interest

The authors declare no conflicts of interest.

## Data Availability

The authors have nothing to report.
